# Complete Genome Sequence of Methylomonas koyamae LM6, a Potential Aerobic Methanotroph

**DOI:** 10.1128/MRA.01544-19

**Published:** 2020-02-06

**Authors:** Dae-Hee Lee, Lavanya Madhavaraj, Gui Hwan Han, Hyewon Lee, Seung-Goo Lee, Si Wouk Kim

**Affiliations:** a Synthetic Biology and Bioengineering Research Center, Korea Research Institute of Bioscience and Biotechnology, Daejeon, Republic of Korea; b Department of Biosystems and Bioengineering, KRIBB School of Biotechnology, University of Science and Technology, Daejeon, Republic of Korea; c Department of Environmental Engineering, Chosun University, Gwangju, Republic of Korea; d Center for Industrialization of Agriculture and Livestock Microorganisms, Jeongeup, Republic of Korea; University of Southern California

## Abstract

Methylomonas koyamae LM6 is a potential methanotrophic bacterium of interest for methane bioconversion. Here, we report the complete genome sequence of M. koyamae LM6, which contains 4,337 predicted open reading frames on one chromosome (4,894,002 bp) and one plasmid (186,658 bp), with genes involved in methane oxidation.

## ANNOUNCEMENT

The genus *Methylomonas*, in the class *Gammaproteobacteria*, contains type I methanotrophs (1). *Methylomonas* species play a key role in mitigating greenhouse gas emissions (2, 3) because they utilize methane as the only source of carbon and energy (4). Recently, they were employed to convert an overabundance of methane into economical fuels and chemicals (2, 5, 6). Here, we report the complete genome sequence of Methylomonas koyamae LM6.

M. koyamae LM6 was isolated from a rice paddy field in the Republic of Korea following a reported method (7). Based on the 16S rRNA gene sequence analysis, the isolate is most closely related to M. koyamae JCM 16701 (>99% similarity) (8). For genome sequencing, M. koyamae LM6 was grown at 30°C in a low-salt mineral medium (9) with a methane/air mixture (6:4 [vol/vol]). Genomic DNA was extracted using the i-genomic BYF DNA extraction minikit (iNtRON Biotechnology). A standard PacBio library with average 20-kb inserts was prepared using the RS II SMRTbell template preparation kit v1.0 (Pacific Biosciences) and sequenced using PacBio RS II single-molecule real-time (SMRT) sequencing technology (Pacific Biosciences). The 72,022 postfiltered reads, with an *N*_50_ value of 21,348 bp, were assembled *de novo* using the Hierarchical Genome Assembly Process (HGAP) pipeline of SMRT Analysis v2.3.0 (10), with genome coverage of 187×. The following parameters were adopted for genome assembly: minimum subread length, 100, minimum polymerase read quality, 0.80, and minimum polymerase read length, 500 (for filtering); compute minimum seed read length, true, and total alignment candidates, 24 (for preassembly) minReadLength, 200, maxScore, 1,000, and maxLCPLength, 16 (for BLASR analysis); and target coverage, 30, overlapper error rate, 0.06, overlapper min length, 40, and overlapper k-mer, 14 (for assembly). To troubleshoot *de novo* assembly, *in silico* resequencing with the BridgeMapper protocol of SMRT Portal (10) was performed twice on the circular chromosome and plasmid of M. koyamae LM6. The estimated finishing quality (consensus concordance value) was QV60. The consensus concordance between the raw reads and assembled sequences was determined with the Quiver algorithm built-in single-molecule real-time (SMRT) pipeline (10, 11). Coding DNA sequences were predicted with Prodigal v2.6.3 (12). Signal peptides and transmembrane domains were predicted using SignalP v4.1 (13) and TMHMM v2.0 (14), respectively. BLAST searches were performed against the UniProt (15), Pfam (16), and Clusters of Orthologous Groups (COG) (17) databases to functionally annotate predicted genes. rRNAs, tRNAs, and miscellaneous features were predicted using RNAmmer (18), tRNAscan-SE (19), and Rfam v12.0 (20), respectively. Default parameters were used for all software unless otherwise specified.

The genome of M. koyamae LM6 consists of one circular chromosome of 4,894,002 bp and one plasmid of 186,658 bp, with an average G+C content of 56.23%. A total of 4,490 genes were predicted, of which 4,337 are predicted open reading frames, 3 are rRNA gene operons (16S, 23S, and 5S), and 48 are tRNAs. M. koyamae LM6 harbors two operons encoding particulate methane monooxygenases (*pmoABC* and *pmoCAB*), whereas soluble methane monooxygenase operons were not found. LM6 also possesses gene clusters for methanol dehydrogenases (*mxaFJGIRPSACKL*).

### Data availability.

The genome sequence data for M. koyamae LM6 were deposited under GenBank accession numbers CP023669 (chromosome) and CP023670 (plasmid), BioProject accession number PRJNA394621, SRA accession number SRS2358343, and BioSample accession number SAMN07357002.
